# A Multiscale Spatiotemporal Causal Mapping Algorithm for Revealing Neural Network Mechanisms of Transcutaneous Auricular Vagus Nerve Stimulation

**DOI:** 10.1002/hbm.70615

**Published:** 2026-07-22

**Authors:** Weiyi Wang, Ruimin Wang, Pan Lin, Yue Leng, Tianqi Huang, Sheng Ge

**Affiliations:** ^1^ School of Biomedical Engineering Shanghai Jiao Tong University Shanghai China; ^2^ Department of Electrical and Electronic Engineering, Faculty of Science and Engineering Saga University Saga Japan; ^3^ Department of Psychology and Cognition and Human Behavior Key Laboratory of Hunan Province Hunan Normal University Changsha China; ^4^ Key Laboratory of Child Development and Learning Science, Ministry of Education, School of Biological Science and Medical Engineering Southeast University Nanjing China

**Keywords:** brain network, effective connectivity (EC), functional magnetic resonance imaging (fMRI), multiscale functional connectivity (FC), transcutaneous auricular vagus nerve stimulation (taVNS)

## Abstract

Transcutaneous auricular vagus nerve stimulation (taVNS) has shown promise in enhancing cognitive and emotional functions, yet its neural mechanisms remain unclear largely because existing analytical methods cannot characterize multiscale functional connectivity nor reliably infer causal interactions between brain regions in the presence of hemodynamic delays in fMRI signals. To address these limitations, we propose a Multiscale Spatiotemporal Causal Mapping (MSTCM) algorithm that integrates community‐aware multiscale functional connectivity with delay‐compensated causal inference. This design enables MSTCM to characterize multiscale connectivity structure and infer directed information flow with enhanced robustness. In evaluations using simulated fMRI data, MSTCM significantly outperformed seven existing causal inference algorithms across multiple evaluation metrics, including precision, sensitivity, Matthews correlation coefficient (MCC), and area under the receiver operating characteristic curve (AUC). Applied to resting‐state fMRI across four predefined large‐scale cortical networks before and after taVNS, MSTCM revealed that taVNS reduced functional coupling between the left lateral sensorimotor cortex (L‐LSMC) and the right intraparietal sulcus (R‐IPS), increased global efficiency, enhanced causal integration within the salience network (SN), weakened causal connectivity within the dorsal attention network (DAN), and strengthened information flow from DAN to SN. These findings suggest that taVNS may enhance cognitive flexibility and emotional regulation by shifting information processing from exteroceptive toward interoceptive pathways and improving large‐scale network efficiency. Consequently, this study provides not only a novel methodological approach but also new neuroimaging evidence supporting the clinical potential of taVNS.

## Graphical abstract

In this study, we propose a multiscale spatiotemporal causal mapping (MSTCM) algorithm integrating multiscale functional connectivity and delay‐aware causal inference. Applied to resting‐state fMRI data, MSTCM reveals that taVNS reorganizes large‐scale brain networks by enhancing global integration efficiency while shifting information processing from exteroceptive toward more flexible, interoceptive pathways.

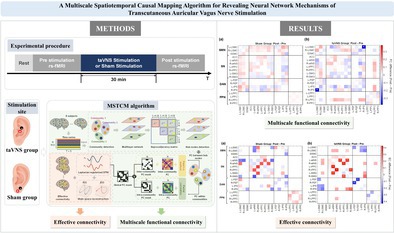

## 1 Introduction

In recent years, noninvasive neuromodulation techniques have attracted growing attention for their potential to enhance cognitive recovery and ameliorate brain dysfunction (Mehta et al. 2024; Zu et al. 2025). Among these approaches, transcutaneous auricular vagus nerve stimulation (taVNS) has emerged as a particularly promising modality owing to its noninvasive, low‐cost, and user‐friendly characteristics, as well as its ability to modulate activity within deep brain regions (Liu et al. 2025, 2020; Toschi et al. 2023). Prior investigations have demonstrated the efficacy of taVNS in enhancing cognitive performance (Sommer et al. 2023), improving emotional regulation (Tan et al. 2023), and modulating the autonomic nervous system (Ludwig et al. 2024). However, its underlying neural mechanisms remain poorly understood, contributing to marked interindividual variability in treatment outcomes and constraining its clinical applicability (Gerges et al. 2024). Thus, elucidating the neural basis of taVNS remains a critical objective in advancing its therapeutic potential.

Recently, the scope of research on taVNS has expanded beyond clinical and behavioral investigations to encompass neuroimaging studies, thereby providing initial evidence for its underlying neurological mechanisms. For instance, Zhang et al. (2021) reported that taVNS modulates thalamocortical circuits in patients with migraine, thereby alleviating headache symptoms; Chen, Tang, et al. (2023) demonstrated that taVNS enhances motor learning by increasing functional connectivity toward the frontal lobe; Poppa et al. (2022) observed that taVNS modulates the insula and its functionally connected regions; and Qi et al. (2025) found that taVNS improves insomnia by altering functional connectivity among the visual, somatosensory, and medial prefrontal cortices. Collectively, these findings highlight the significant neuromodulatory potential of taVNS. Nevertheless, most existing studies rely on analytical approaches that lack a multiscale analytical perspective and do not characterize the directional interactions that underpin taVNS‐induced modulation, thereby limiting a comprehensive understanding of its network‐level effects.

Functional connectivity (FC) is a widely used approach for investigating brain networks and can be broadly classified into linear and nonlinear methods. Linear methods, such as coherence (Smith et al. 2011), partial correlation (Ryali et al. 2012), and canonical correlation analysis (Zhuang et al. 2020) are computationally efficient and require few parameters, making them effective for quantifying synchronous neural relationships. Nonetheless, they are inherently limited in their ability to detect complex interactions and higher‐order topological features. In contrast, nonlinear methods, including mutual information (Motlaghian et al. 2022), phase synchronization (Honari et al. 2021), and Patel's κ conditional dependence (Patel et al. 2006), excel at capturing more intricate, fine‐scale interactions but are often computationally expensive and highly susceptible to noise. The human brain exhibits a modular, small‐world organization, in which dense intra‐community connections enable efficient local cooperation, whereas hub regions facilitate inter‐community integration and exhibit stronger nonlinear features (Bordier et al. 2017; Sporns and Betzel 2016). Consequently, single‐scale FC analyses often fail to capture both intra‐ and inter‐community characteristics simultaneously. Mohanty et al. (2020) further demonstrated that different FC measures can yield divergent network patterns, underscoring that single‐scale methods are insufficient for a comprehensive representation of brain network organization.

To address these limitations, several new FC methods have been proposed. Li, Zhou, et al. (2021) developed BrainGNN, an interpretable graph neural network that integrates ROI‐aware convolutions with ROI‐selection pooling to identify salient regions and community structures; however, it does not provide fine‐grained characterization of multiscale connectivity patterns. Zalesky et al. (2024) employed deep neural networks to optimize structure–function mapping, which improved individualized predictions but lacked multiscale integration. Faskowitz et al. (2020) proposed an edge‐centric representation of functional connectivity that captures overlapping architecture through edge–edge co‐fluctuations; however, it remains correlation‐based and lacks hierarchical integration across multiple connectivity scales. Although these approaches have advanced FC analysis, they largely remain restricted to single‐scale or global features and consequently fail to provide comprehensive multiscale modeling of both intra‐ and inter‐community connectivity.

Additionally, studying the neural regulatory mechanisms of taVNS requires not only focusing on FC but also examining causal interactions. Effective connectivity (EC) characterizes the directional flow of information between brain regions through causal inference, which is crucial for revealing the underlying neural regulatory mechanisms (Danks and Davis 2023). In taVNS research, functional magnetic resonance imaging (fMRI) is commonly employed because of its high spatial resolution. However, the low sampling rate of fMRI and the hemodynamic delay associated with the blood oxygen level‐dependent (BOLD) signal often pose challenges to accurately identifying causal relationships (Chen, Taylor, et al. 2023; Finn et al. 2023). Existing causal analysis methods include Granger causality (GC) (Zhang et al. 2025; Hasanzadeh et al. 2021), dynamic causal modeling (DCM) (Frässle et al. 2021; Wang and Liang 2021), and structural equation modeling (SEM) (Cosio‐Guirado et al. 2022; Garrett et al. 2023). Although these methods have advanced the interpretation of causal relationships among brain regions, they rely on prior model assumptions, require strict data stationarity, and exhibit inherent limitations when applied to nonlinear and high‐dimensional brain systems. More recently, deep learning‐based causal discovery approaches, including recurrent neural network (RNN)‐based methods (Khanna and Tan 2020) and Transformer‐based architectures (Kong et al. 2024), have demonstrated promising capacity for capturing nonlinear temporal dependencies in high‐dimensional time series. Nevertheless, their application to neuroimaging data remains challenging due to the requirement for large training datasets and the imperative for physiological interpretability when interpreting hemodynamic‐confounded signals.

In recent years, convergent cross mapping (CCM) has emerged as an effective tool for analyzing causal relationships in complex systems, as it does not require prior structural assumptions or data stationarity (Sugihara et al. 2012; Deng et al. 2024). However, studies have shown that CCM is sensitive to time delays (Ye et al. 2015), and this sensitivity may reduce the reliability of causal‐direction inference in fMRI data analysis. To address this limitation, researchers have proposed several variants of CCM that account for time delay compensation: Ye et al. (2015) improved causal inference accuracy by explicitly modeling time delays; Martin et al. (2024) introduced a time‐delay selection criterion based on global mutual information maximization; and Isufaj et al. (2025) implemented time‐lag optimization within the cross‐mapping process for enhanced causal detection. Nevertheless, these methods primarily focus on time‐domain processing and have not fully considered the coupling between spatial topology and information‐propagation delays.

Despite growing evidence of the neuromodulatory effects of taVNS, two fundamental challenges continue to limit a deeper mechanistic understanding. First, the modular and hierarchical organization of the brain is rarely incorporated into analytical workflows, making it difficult to characterize how taVNS reshapes network architecture across multiple spatial scales. Second, accurately identifying directed neural interactions in fMRI remains challenging because hemodynamic delays obscure the true temporal structure of causal relationships. Together, these challenges hinder efforts to delineate the multiscale and directional reorganization of large‐scale networks induced by taVNS. To advance this line of inquiry, this study introduces a causal modeling algorithm that integrates multiscale functional connectivity with delay‐aware causal inference, enabling a systematic characterization of taVNS‐induced modulations in network structure and information flow across four predefined large‐scale cortical networks. This work not only provides theoretical support for the clinical translation of taVNS but also introduces a novel methodological perspective for brain‐network analysis in the field of neuromodulation.

## 2 Methods and Materials

To systematically explore the neural regulatory mechanisms of taVNS from a brain network perspective, we propose a Multiscale Spatiotemporal Causal Mapping (MSTCM) algorithm. This algorithm integrates multiscale functional connectivity and effective connectivity into a unified approach for characterizing taVNS‐induced network regulation. MSTCM comprises two modules: Spatial Multiscale Functional Connectivity (SM‐FC) and Spatiotemporal Causal Mapping (STCM), forming a comprehensive modeling pipeline from functional connectivity estimation to causal inference.

Subsequently, to rigorously validate the algorithm's effectiveness and scientific soundness, we first evaluated the performance of the SM‐FC and STCM modules on simulated fMRI data. We then compared the overall MSTCM framework with several representative causal inference methods using the same simulated datasets. Finally, MSTCM was applied to empirical fMRI data from a taVNS experiment. This application aimed to reveal changes in brain networks at the levels of both functional and effective connectivity before and after the electrical stimulation, thereby offering a comprehensive interpretation of the neural regulatory mechanisms of taVNS.

### 2.1 MSTCM

**FIGURE 1:**
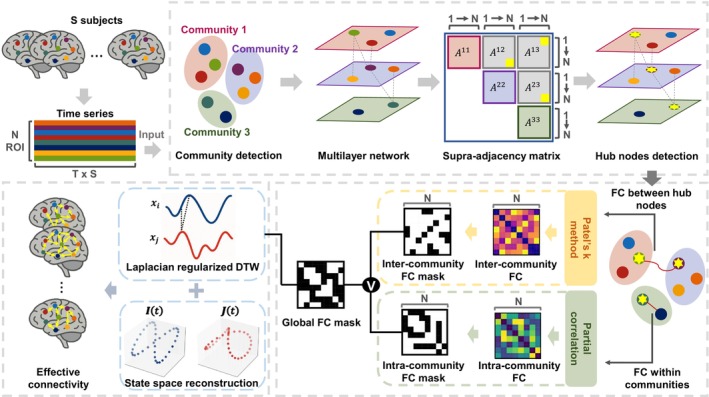
Overall workflow of the proposed MSTCM algorithm.

The overall workflow of the proposed MSTCM algorithm is illustrated in Figure 1. First, multiscale functional connectivity is estimated by integrating linear and nonlinear methods, based on the modular organization of the brain's small‐world network. Next, guided by the estimated FC, causal relationships between nodes are quantified in terms of both direction and strength using spatiotemporal registration and cross‐mapping, thereby elucidating the information flow pathways and brain network mechanisms underlying taVNS stimulation.

#### 2.1.1 SM‐FC Module

Let $x\in {\mathbb{R}}^{N\times T}$ denote the ROI time series matrix for a single subject, where N and T represent the number of ROIs and the time series length, respectively. To obtain a group‐level connectivity pattern and improve statistical stability, the time series matrices from all subjects are concatenated along the time axis, yielding $X=\left[x^{\left(1\right)},x^{\left(2\right)},\ldots ,x^{\left(S\right)}\right]\in {\mathbb{R}}^{N\times \left(T\cdot S\right)}$, where S denotes the number of subjects. The group‐level functional connectivity matrix $W\in {\mathbb{R}}^{N\times N}$ is computed using Pearson correlation, with diagonal elements $W_{i,i}=0$ to avoid self‐connections. The Louvain algorithm is applied for community detection (Blondel et al. 2008), and negative weights in W are symmetrized (Rubinov and Sporns 2011). The modularity score is defined as:

(1) $$Q=\frac{1}{2m}\sum_{i,j}\left(W_{\mathrm{ij}}-\frac{k_{i}k_{j}}{2m}\right)\delta \;\left(C\left(i\right),C\left(j\right)\right),$$

where $k_{i}=\sum_{j}W_{\mathrm{ij}}$ represents the total weight of all edges connected to node i, and $m=\frac{1}{2}\sum_{i,j}W_{\mathrm{ij}}$ is the total edge weight. $\delta \;\left(C\left(i\right),C\left(j\right)\right)=1$ if nodes i and j belong to the same community, and 0 otherwise. $C\left(i\right)\in \left\{1,2,\ldots ,G\right\}$ denotes the community label of node i, where G is the total number of communities.

To identify hub nodes across communities, a multilayer network modeling strategy is employed to construct a supra‐adjacency matrix $A\in {\mathbb{R}}^{\left(N\cdot G\right)\times \left(N\cdot G\right)}$, representing both intra‐ and inter‐community connections. The matrix consists of G×G submodules, and for each pair of communities $C_{k}$ and $C_{l}$, where $k,l\in \left\{1,2,\ldots ,G\right\}$, the corresponding submodule $A^{\mathrm{kl}}$ is defined as:

(2) $$A_{\left(k-1\right)N+i,\;\left(l-1\right)N+j}^{\mathrm{kl}}=W_{\mathrm{ij}},$$

where i and j represent nodes from communities $C_{k}$ and $C_{l}$ respectively. In A, diagonal blocks represent intra‐community connections, while the off‐diagonal blocks represent inter‐community connections. The connectivity strength of hub nodes is quantified as:

(3) $$d_{\text{inter}}\left(i^{\prime }\right)=\sum_{j^{\prime }\neq i^{\prime }}A_{i^{\prime }j^{\prime }},\;i^{\prime },j^{\prime }\in \left\{1,2,\ldots ,N\cdot G\right\},$$

where $i^{\prime }$ and $j^{\prime }$ are the indices of node i and j in A, and modulo operations are used to map $i^{\prime }$ and $j^{\prime }$ back to the original node indices $i,j\in \left\{1,2,\ldots ,N\right\}$:

(4) $$i=\mathrm{mod}\left(i^{\prime }-1,N\right)+1.$$

Nodes with inter‐layer connectivity strength exceeding the quantile threshold α are considered hub nodes across modules.

Let $P\in {\mathbb{R}}^{N\times N}$ represent intra‐community connectivity. For community $C_{k}$, the time series of all nodes is $x_{C_{k}}\in {\mathbb{R}}^{N_{k}\times T}$, where $N_{k}$ is the number of nodes in $C_{k}$. Partial correlation is used to quantify the functional connectivity $\rho_{\mathrm{mn}}^{k}$ between nodes m and n in $C_{k}$. The global intra‐community matrix P is constructed as:

(5) $$P_{\mathrm{mn}}=\left\{\begin{array}{ll} \rho_{\mathrm{mn}}^{k}, & \text{if}\;m,n\in C_{k},\\ 0, & \text{otherwise}. \end{array}\right.$$

To identify statistically significant connections, surrogate time series were generated using the Iterative Amplitude Adjusted Fourier Transform (IAAFT) algorithm, forming a null distribution (DSouza et al. 2018). A hypothesis test at p<0.05 yields a binary intra‐community matrix $M^{\text{intra}}\in {\left\{0,1\right\}}^{N\times N}$.

To capture nonlinear inter‐community interactions, Patel's κ conditional dependence is applied to hub nodes (Patel et al. 2006). Let V be the set of hub nodes, and $N_{v}=\;|V|$ represent their number. The corresponding time series is $x_{V}\in {\mathbb{R}}^{N_{v}\times T}$; normalize it to $m_{V}\in {\mathbb{R}}^{N_{v}\times T}$ (with values below the 10th percentile set to 0, above the 90th percentile set to 1, and intermediate values linearly mapped to $\left[0,1\right]$). Binarization is then performed with threshold θ=0.25, producing activation matrix $B\in {\left\{0,1\right\}}^{N_{v}\times T}$, where $B_{\mathrm{it}}=1$ denotes node i being active at time t. Patel's κ conditional dependence yields inter‐community $q_{\mathrm{ab}}$ between hub nodes a and b (Patel et al. 2006), forming:

(6) $$Q_{\mathrm{ab}}=\left\{\begin{array}{ll} q_{\mathrm{ab}}, & \text{if}\;a,b\in V,\\ 0, & \text{otherwise}. \end{array}\right.$$

The same surrogate testing (IAAFT, p<0.05) produces the binary inter‐community matrix $M^{\text{inter}}\in {\left\{0,1\right\}}^{N\times N}$. The final functional connectivity mask is:

(7) $$M_{\mathrm{ij}}=\max\;\left(M_{\mathrm{ij}}^{\text{intra}},M_{\mathrm{ij}}^{\text{inter}}\right),\;\forall i,j\in \left\{1,2,\ldots ,N\right\},$$

where $M_{\mathrm{ij}}=1$ indicates a significant functional connection between nodes i and j. The global functional connectivity matrix $F\in {\mathbb{R}}^{N\times N}$ is defined as:

(8) $$F_{\mathrm{ij}}=\left\{\begin{array}{ll} P_{\mathrm{ij}}, & \text{if}\;P_{\mathrm{ij}}\neq 0,\\ Q_{\mathrm{ij}}, & \text{if}\;Q_{\mathrm{ij}}\neq 0,\\ 0, & \text{otherwise}. \end{array}\right.$$

The fusion strategy in Equations (7) and (8) implements a threshold‐based priority union: a connection is retained if it is statistically significant at either the intra‐ or inter‐community scale. Because $P_{\mathrm{ij}}$ and $Q_{\mathrm{ij}}$ are defined on mutually exclusive node pairs by construction, with partial correlation applied to intra‐community pairs and Patel's κ applied to inter‐community hub pairs, each connection is unambiguously assigned the metric appropriate to its spatial scale, ensuring that both linear intra‐community synchrony and nonlinear inter‐community interactions are captured within a unified connectivity representation.

#### 2.1.2 STCM Module

Based on the functional connectivity mask M, the Laplacian matrix $L\in {\mathbb{R}}^{N\times N}$ is constructed as:

(9) L=R−M,

where $R\in {\mathbb{R}}^{N\times N}$ is the degree matrix with $R_{\mathrm{ii}}=\sum_{j}M_{\mathrm{ij}}$. The Laplacian matrix L serves as a topological regularization term, introducing spatial structural constraints into causal estimation. Considering that fMRI signals are affected by hemodynamic effects and thus exhibit temporal misalignments, STCM incorporates dynamic time warping (DTW) with Laplacian regularization. The distance matrix $D_{\mathrm{ij}}\in {\mathbb{R}}^{\left(T+1\right)\times \left(T+1\right)}$ between nodes i and j is:

(10) $$\begin{array}{l} D\left(r,s\right)=\min\left\{D\left(r-1,s\right),D\left(r,s-1\right),D\left(r-1,s-1\right)\right\}\\ +{\left(x_{i}\left(r-1\right)-x_{j}\left(s-1\right)\right)}^{2}+\gamma L_{\mathrm{ij}}, \end{array}$$

where $r,s\in \left[1,T+1\right]$, γ is the regularization parameter, and $L_{\mathrm{ij}}$ is the Laplacian weight between i and j. $D\left(r,s\right)$ represents the cumulative distance when aligning the *r*‐th time point of $x_{i}$ with the *s*‐th time point of $x_{j}$. By backtracking from $D\left(T+1,T+1\right)$ to $D\left(1,1\right)$, the minimal cumulative distance path is obtained, denoted as:

(11) $$P_{\mathrm{ij}}={\left\{[d_{i}\left(k\right),d_{j}\left(k\right)]\right\}}_{k=1}^{K}$$

where $P_{\mathrm{ij}}$ denotes the optimal warping path between $x_{i}$ and $x_{j}$, K is the length of this path, and each pair $\left[d_{i}\left(k\right),d_{j}\left(k\right)\right]$ indicates that the $d_{i}\left(k\right)$‐th time point of $x_{i}$ is aligned with the $d_{j}\left(k\right)$‐th time point of $x_{j}$. This path provides the temporal correspondences for subsequent cross‐mapping.

For each node, delay embedding reconstructs the state space. For time point $t\in \left[T_{\min},T\right]$, where $T_{\min}=1+\left(E-1\right)\tau$ denotes the earliest valid time index for delay embedding, the delay‐embedded vectors are:

(12) $$I\left(t\right)=\left[x_{i}\left(t\right),x_{i}\left(t-\tau \right),\ldots ,x_{i}\left(t-\left(E-1\right)\tau \right)\right],$$

(13) $$J\left(t\right)=\left[x_{j}\left(t\right),x_{j}\left(t-\tau \right),\ldots ,x_{j}\left(t-\left(E-1\right)\tau \right)\right],$$

where E is embedding dimension and τ is the time delay. The reconstructed state spaces are:

(14) $$I_{m}=\left\{I\left(T_{\min}\right),I\left(T_{\min}+1\right),\ldots ,I\left(T\right)\right\},$$

(15) $$J_{m}=\left\{J\left(T_{\min}\right),J\left(T_{\min}+1\right),\ldots ,J\left(T\right)\right\}.$$

To evaluate the causal influence, the STCM module employs an improved cross‐mapping strategy. If node i influences node j, then the state space of $x_{j}$ should contain information about $x_{i}$. Thus, $x_{i}$ can be reconstructed from $J_{m}$ (Sugihara et al. 2012). In the algorithm, to incorporate spatial topological constraints, the aforementioned alignment path is utilized to identify time points in $x_{i}$ corresponding to the nearest neighbor embeddings of $x_{j}$, thereby establishing predictive correspondences in the state space.

Based on the above idea, $J_{m}$ is used to predict $x_{i}\left(t\right)$. The specific steps are as follows: In $J_{m}$, find the E+1 nearest neighbors of $J\left(t\right)$, whose corresponding time points in $x_{j}$ are denoted as $\left\{n_{1},n_{2},\ldots ,n_{E+1}\right\}$, with corresponding Euclidean distances $\left\{d_{1},d_{2},\ldots ,d_{E+1}\right\}$. Using the alignment path, each $n_{p}$ is mapped to the time indices in $x_{i}$ that are aligned with it:

(16) $$S_{p}=\left\{d_{i}\left(k\right)\;|\;d_{j}\left(k\right)=n_{p}\right\}$$

where $S_{p}$ denotes the set of time points in $x_{i}$ aligned to $n_{p}$ along the DTW path. The predicted value of $x_{i}\left(t\right)$ from $J_{m}$, denoted as ${\left.x_{i}^{*}\left(t\right)\right|}_{J_{m}}$, is computed as:

(17) $$u_{p}=\exp\left(-\frac{d_{p}}{d_{1}}\right),$$

(18) $$w_{p}=\frac{u_{p}}{\sum_{q=1}^{E+1}u_{q}},$$

(19) $${\bar{x}}_{i}\left(n_{p}\right)=\frac{1}{|S_{p}|}\sum_{q\in S_{p}}x_{i}\left(q\right),$$

(20) $${\left.x_{i}^{*}\left(t\right)\right|}_{J_{m}}=\sum_{p=1}^{E+1}w_{p}\;{\bar{x}}_{i}\left(n_{p}\right),$$

where $d_{p}$ is the Euclidean distance to the *p*‐th nearest neighbor of $J\left(t\right)$, and ${\bar{x}}_{i}\left(n_{p}\right)$ is the average of all $x_{i}$ values aligned to the *p*‐th neighbor via the DTW path, providing a single representative value when DTW yields one‐to‐many temporal correspondences. Similarly, $x_{j}\left(t\right)$ can be predicted from the mapping of $x_{i}$, denoted as ${\left.x_{j}^{*}\left(t\right)\right|}_{I_{m}}$. The reconstructed time series ${\left.x_{i}^{*}\right|}_{J_{m}}$ is obtained by stacking the pointwise estimates ${\left.x_{i}^{*}\left(t\right)\right|}_{J_{m}}$ across all valid time points. The causal strength from node i to node j is measured by the Pearson correlation between the reconstructed and actual values:

(21) $$\rho_{i\to j}=\text{corr}\left({\left.x_{i},\;x_{i}^{*}\right|}_{J_{m}}\right),$$

where $\rho_{i\to j}$ denotes the Pearson correlation coefficient. If node i causally drives node j, then the historical states of $x_{j}$ should encode information about $x_{i}$, enabling accurate reconstruction through cross‐mapping.

The SM‐FC module is computed once at the group level, with complexity $O\left(U\left(N^{2}\cdot T\cdot S+\frac{N^{3}}{G^{2}}\right)\right)$, where U is the number of IAAFT surrogate realizations; the $N^{2}\cdot T\cdot S$ term dominates when $T\cdot S\gg \frac{N}{G^{2}}$. The STCM module runs per subject on the $\eta_{M}\cdot N^{2}$ retained node pairs, with complexity $O\left(S\cdot \eta_{M}\cdot N^{2}\cdot T^{2}\right)$, where $\eta_{M}$ is the mask density. The overall complexity is thus $O\left(U\left(N^{2}\cdot T\cdot S+\frac{N^{3}}{G^{2}}\right)+S\cdot \eta_{M}\cdot N^{2}\cdot T^{2}\right)$.

### 2.2 Simulated fMRI Data and Comparison Algorithms

To validate the proposed MSTCM algorithm, we first evaluated the SM‐FC and STCM modules and subsequently compared the performance of MSTCM with seven classical or emerging effective connectivity algorithms using the Smith simulated fMRI dataset (Smith et al. 2011). The Smith dataset simulates resting‐state fMRI time series based on predefined small‐world network topologies. Specifically, neural activity is generated through dynamic causal modeling (DCM), and the corresponding hemodynamic responses are estimated via the nonlinear balloon model, ultimately producing resting‐state fMRI signals. In these networks, nodes correspond to regions of interest (ROIs), and the connections between the nodes form small‐world topologies. For performance evaluation, 10 representative data samples were selected from the Smith dataset, each comprising 50 virtual subjects. These data differed in network scale, noise level, and perturbation factors that emulated realistic experimental variability, while session duration (10 min), repetition time (TR = 3.0 s), and the standard deviation of the hemodynamic response function (HRF) delay (0.5 s) were kept constant across all simulations to ensure comparability.

**FIGURE 2:**
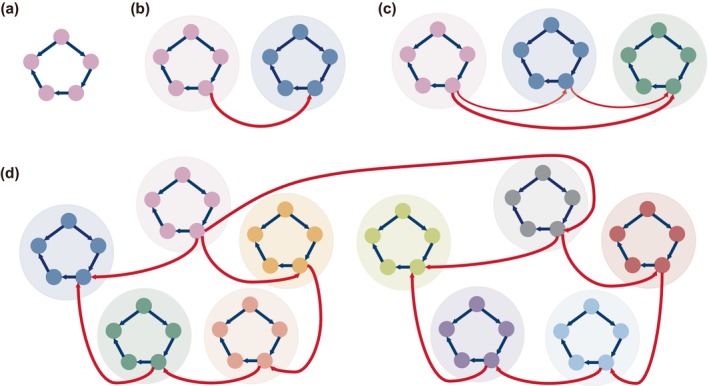
Schematic illustration of the topological structure of the Smith simulated fMRI data at different node scales. Red arrows indicate inter‐module connections, and blue arrows indicate intra‐module connections. ((a–d) correspond to Sim1–Sim4, respectively).

Sim1–Sim4 represented networks with increasing numbers of nodes (5, 10, 15, and 50, respectively), allowing evaluation of the algorithm's scalability from small‐ to large‐scale networks, as illustrated in Figure 2. Sim5–Sim10 introduced additional complexities to assess the algorithm's robustness under challenging conditions. Specifically, Sim5 incorporated shared inputs, in which multiple nodes were driven by the same external source. This setting simulates a realistic scenario where unmodeled neuronal or sensory inputs simultaneously influence several observed regions, producing correlated activity that can obscure true causal relationships. Sim6 introduced a global mean confound by adding a uniform signal component across all nodes, simulating physiological noise or scanner drift. Sim7 simulated inaccurate ROI definitions by mixing each node's BOLD signal with those from other regions, thereby mimicking spatial overlap or functionally mismatched atlas‐based ROIs. Sim8 implemented cyclic connections to examine the model's capacity to detect feedback interactions. Sim9 increased the intrinsic connection strength between nodes, thereby challenging the algorithm's stability under conditions of high coupling. Finally, Sim10 incorporated nonstationary connection strengths that varied over time, reflecting the dynamic fluctuations often observed in real fMRI networks.

These diverse simulation conditions provided a comprehensive framework for evaluating the MSTCM algorithm's performance across different network sizes, noise levels, and connectivity patterns. By encompassing both controlled and perturbed conditions, this design enabled a rigorous assessment of the algorithm's scalability, sensitivity, and robustness relative to other causal inference methods.

The comparison algorithms include large‐scale Granger causality (lsGC) (DSouza et al. 2017), MVARp (Gilson et al. 2017), mutual connectivity analysis with local models (MCA‐LM) (DSouza et al. 2018), convergent cross sorting (CCS) (Breston et al. 2021), FPCMCI (Castri et al. 2023), Stable Differentiable Causal Discovery (SDCD) (Nazaret et al. 2023), and Tangent Space Causal Inference (TSCI) (Butler et al. 2024). The default parameter configurations reported in the respective studies were adopted: lsGC (cmp=4, ARorder=2, normalize=1); MVARp (nperm=1000, a=0.05); MCA‐LM (param=3, norm=1); CCS (lag=0, τ=1, dim=4); FPCMCI (f_a=0.1, pcmci_a=0.05, lag=1); and TSCI (θ=0.5, ${\mathrm{fnn}}_{\tau }=0.005$). SDCD does not require parameter inputs. The proposed MSTCM algorithm is relatively insensitive to parameter selection; therefore, we used commonly adopted settings for state‐space reconstruction methods: α=0.8, θ=0.25, τ=1, E=4, γ=0.1. Performance was evaluated using precision, sensitivity, the Matthews correlation coefficient (MCC), and the area under the receiver operating characteristic curve (AUC).

### 2.3 taVNS Experiment

#### 2.3.1 taVNS Experimental Paradigm

The experiment was approved by the Ethics Committee of Jiangsu Provincial People's Hospital (ethical approval no. 2023‐SR‐658). All participants provided written informed consent prior to participation. Because the goal of this study is to elucidate the foundational network mechanisms through which taVNS modulates large‐scale brain networks, experiments were conducted in healthy adults. Studying healthy subjects enables the observation of mechanistic effects without the confounding influences of disease‐related network alterations or medication effects.

A total of 34 healthy, right‐handed subjects were recruited and randomly assigned to two groups: the taVNS group (17 participants; 7 male/10 female; mean age 22.82±1.59 years, range 18–25) and the Sham group (17 participants; 7 male/10 female; mean age 22.59±2.03 years, range 18–25). No significant between‐group differences were found in age ($t\left(32\right)=0.38$, p=0.71, independent‐samples *t*‐test) or sex distribution. Based on a post hoc sensitivity analysis performed with G*Power 3.1.9.7 (Faul et al. 2009), the statistical power $\left(1-\beta \right)$ was estimated to be 0.85, which exceeds the commonly accepted threshold of 0.80 and indicates that the sample size was adequate to detect large within‐subject effects. All participants had no history of neurological or psychiatric disorders, no metallic implants, and had not taken any medications affecting the nervous system within 1 week prior to the experiment. Electrical stimulation was delivered using a transcutaneous electrical nerve stimulation device (SDZ‐III, Hwato, Suzhou, China).

In the taVNS group, electrical stimulation was delivered to the left cymba concha, thereby activating the afferent pathway of the vagus nerve (Frangos et al. 2015; Butt et al. 2020). In the Sham group, stimulation was applied to the left earlobe, a region where electrical stimulation does not elicit vagus nerve activation (Badran et al. 2018). The electrode spacing was 5 mm, and the stimulation frequency was 20 Hz. The current intensity was individually calibrated using the Method of Limits (Thompson et al. 2021). Specifically, the intensity was gradually increased from a low level until the participant reported discomfort (recorded as $I_{\text{high}}$); it was then slightly exceeded before being decreased until the participant reported a strong sensation that was intense but no longer uncomfortable (recorded as $I_{\mathrm{low}}$). This process was repeated three times, and the mean of the six measurements was used as the individualized stimulation intensity. The actual stimulation intensity was 5.36±0.37 mA (range: 4.8–5.9 mA) in the taVNS group and 5.26±0.36 mA (range: 4.6–5.9 mA) in the Sham group, with no significant between‐group difference ($t\left(32\right)=0.74$, p=0.47, independent‐samples *t*‐test).

**FIGURE 3:**
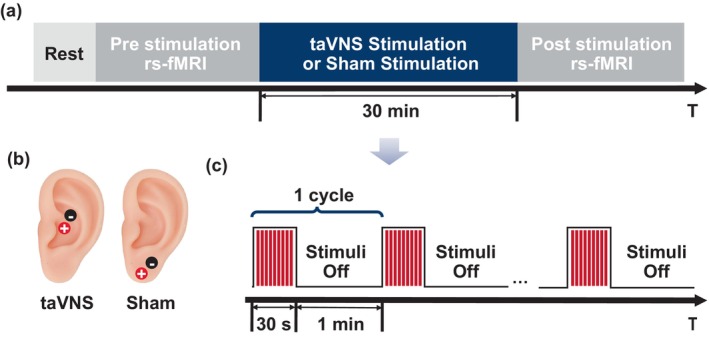
Experimental paradigm. (a) Overall experimental procedure; (b) Stimulation sites for the taVNS group (cymba concha) and the Sham group (earlobe). Red dots indicate the positive electrodes, and black dots indicate the negative electrodes.

The experimental procedure consisted of three stages: acquisition of a pre‐stimulation resting‐state fMRI scan, a 30‐min electrical stimulation intervention, and acquisition of a post‐stimulation resting‐state fMRI scan (Zhang et al. 2024a, 2024b). The stimulation phase included 20 cycles, each comprising 30 s of stimulation followed by a 1‐min rest interval, yielding a total duration of 30 min. Before the experiment, all participants rested for 5 min to stabilize physiological conditions, after which baseline fMRI data were acquired. Immediately following the stimulation phase, a second resting‐state fMRI scan was performed, as illustrated in Figure 3.

#### 2.3.2 fMRI Data Collection and Preprocessing

Resting‐state fMRI data were acquired using a Siemens Skyra 3.0 T scanner. During the scans, participants were instructed to keep their eyes closed, remain still, and avoid falling asleep, while their heads were stabilized with foam padding to minimize motion artifacts. Earplugs were provided to attenuate scanner noise. Ten minutes of resting‐state fMRI data were collected using a T2‐weighted single‐shot gradient‐echo planar‐imaging sequence with the following parameters: repetition time (TR) = 2530 ms, echo time (TE) = 30 ms, flip angle = $90^{{}^{\circ}}$, 41 axial slices, slice thickness = 3.5 mm, field of view (FOV) = 256 mm, matrix size = 64×64, 240 volumes. High‐resolution T1‐weighted structural images were acquired using a magnetization‐prepared rapid acquisition gradient‐echo (MPRAGE) sequence, with TR = 1900 ms, TE = 2.45 ms, flip angle = $7^{{}^{\circ}}$, matrix size = 256×256, 176 sagittal slices, and slice thickness = 1 mm.

fMRI data preprocessing was conducted using the CONN toolbox (version 22v2407) (Whitfield‐Gabrieli and Nieto‐Castanon 2012). The preprocessing steps included discarding the first five volumes, realignment and motion correction, slice‐timing correction, outlier detection, denoising, spatial normalization, and smoothing with an 8 mm full‐width at half‐maximum (FWHM) Gaussian kernel. During the denoising step, physiological and spurious noise was removed using the anatomical CompCor (aCompCor) approach, in which the top five principal components extracted from white matter and cerebrospinal fluid masks were regressed from the BOLD signal (Behzadi et al. 2007). Additional denoising regressors included six rigid‐body motion parameters and their first‐order temporal derivatives. Band‐pass filtering (0.008–0.09 Hz) was subsequently applied to further suppress low‐frequency drift and residual physiological noise. Although dedicated cardiac and respiratory recordings were not acquired during scanning, aCompCor has been validated as an effective surrogate for RETROICOR‐based physiological noise removal in the absence of external physiological data (Chai et al. 2012). Subsequently, ROI time series were extracted from the preprocessed functional images (Nieto‐Castanon 2020). The analysis included all ROIs from four large‐scale cortical networks, comprising 18 ROIs in total: the sensorimotor network (SMN), salience network (SN), dorsal attention network (DAN), and frontoparietal network (FPN). These networks were chosen to represent four functional domains relevant to the present study: sensorimotor processing, salience/interoceptive processing (Seeley et al. 2007), externally oriented attention (Corbetta and Shulman 2002), and flexible cognitive control (Marek and Dosenbach 2018). The network affiliations, ROI names, abbreviations, and MNI coordinates are summarized in Table 1.

**TABLE 1:** ROIs used in this study, their abbreviations, and MNI coordinates.

Brain network	ROI name	Abbreviation	MNI coordinates (mm)		
			*x*	*y*	*z*
Sensorimotor network, SMN	Left lateral sensorimotor cortex	L‐LSMC	−55	−12	29
	Right lateral sensorimotor cortex	R‐LSMC	56	−10	29
	Superior sensorimotor cortex	SSMC	0	−31	67
Salience network, SN	Anterior cingulate cortex	ACC	0	22	35
	Left anterior insula	L‐aINS	−44	13	1
	Right anterior insula	R‐aINS	47	14	0
	Left rostral prefrontal cortex	L‐rPFC	−32	45	27
	Right rostral prefrontal cortex	R‐rPFC	32	46	27
	Left supramarginal gyrus	L‐SMG	−60	−39	31
	Right supramarginal gyrus	R‐SMG	62	−35	32
Dorsal attention network, DAN	Left frontal eye field	L‐FEF	−27	−9	64
	Right frontal eye field	R‐FEF	30	−6	64
	Left intraparietal sulcus	L‐IPS	−39	−43	52
	Right intraparietal sulcus	R‐IPS	39	−42	54
Frontoparietal network, FPN	Left lateral prefrontal cortex	L‐LPFC	−43	33	28
	Left posterior parietal cortex	L‐PPC	−46	−58	49
	Right lateral prefrontal cortex	R‐LPFC	41	38	30
	Right posterior parietal cortex	R‐PPC	52	−52	45

## 3 Results

### 3.1 Results on Simulated Data

#### 3.1.1 Performance of the SM‐FC Module

**FIGURE 4:**
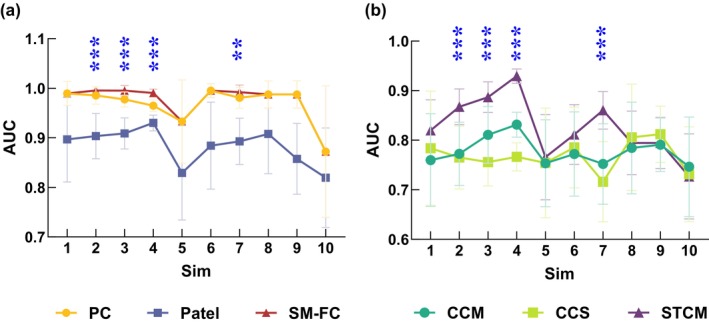
SM‐FC and STCM performance on Smith simulated data. (a) AUC comparison between SM‐FC and two baseline methods; (b) AUC comparison between STCM and two baseline methods (Error bars represent standard deviation, **p<0.01, ***p<0.001; Friedman test followed by Dunn's multiple comparisons. Asterisks are displayed only when the proposed method significantly outperforms all baseline methods at the corresponding significance level).

Using the Smith simulated data, we first evaluated functional connectivity and compared the performance of the proposed SM‐FC module with two single‐scale baseline methods: Pearson correlation (PC) and Patel's κ conditional dependence (Patel et al. 2006). Statistical significance was assessed using the Friedman test, followed by Dunn's post hoc test for multiple comparisons (Demšar 2006). The results showed that SM‐FC significantly outperformed Patel's κ conditional dependence across all simulated data (Sim1–Sim9: p<0.001; Sim10: p<0.01). When compared with the PC method, SM‐FC showed a highly significant improvement in Sim2, Sim3, and Sim4 (with increasing node numbers) (p<0.001), and also demonstrated significant superiority in Sim7 (which included lower quality ROIs) (p<0.01). In the remaining datasets, SM‐FC performed similarly to PC, as shown in Figure 4a. Overall, SM‐FC significantly outperformed single‐scale methods in functional connectivity estimation, especially in large‐scale networks and under noisy conditions.

#### 3.1.2 Performance of the STCM Module

To evaluate the proposed STCM module in inferring causal interactions, we compared its performance with two baseline methods: Convergent Cross Mapping (CCM) (Sugihara et al. 2012) and Convergent Cross Sorting (Breston et al. 2021). Statistical significance was assessed using the Friedman test, followed by Dunn's post hoc test for multiple comparisons. As shown in Figure 4b, STCM significantly outperformed both CCM and CCS in Sim2, Sim3, Sim4, and Sim7 (p<0.001). These findings suggest that the STCM method effectively reduces causal inference bias resulting from the low temporal resolution of fMRI data and the hemodynamic delay inherent in the BOLD signal.

#### 3.1.3 Overall Performance of the MSTCM Model

**FIGURE 5:**
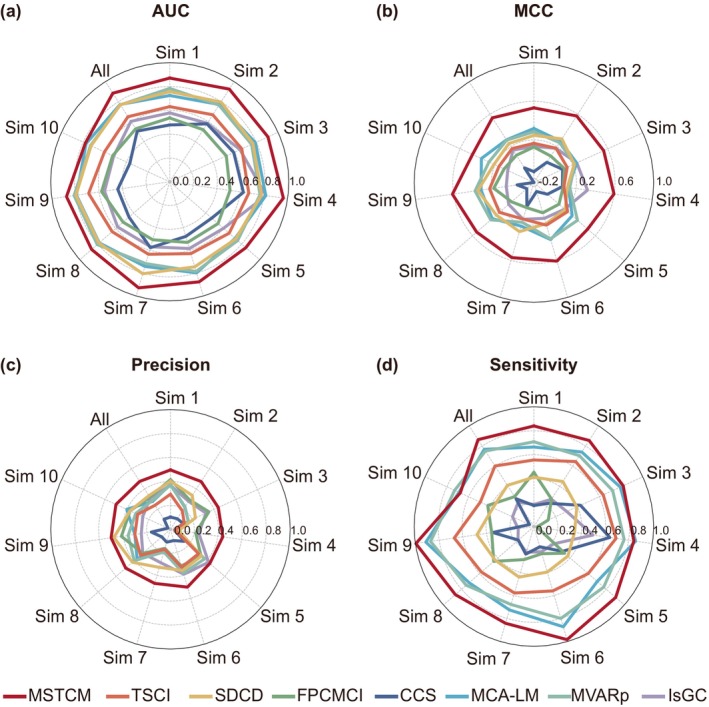
Overall performance and stability comparison of causal inference algorithms on the simulated data. (a) AUC, (b) MCC, (c) precision, (d) sensitivity. The radar chart illustrates the comparison results across 10 simulated conditions (Sim1–Sim10) and the aggregated overall performance (All).

As shown in Figure 5, we compared the performance of MSTCM with seven baseline causal inference methods across 10 simulated datasets (Sim1–Sim10) and the overall aggregated data (All). Statistical significance was assessed using the Friedman test, followed by Dunn's post hoc test for multiple comparisons. Complete numerical results for all methods and conditions are provided in Table S1. The results indicate that MSTCM achieved the highest AUC, MCC, precision, and sensitivity across nearly all simulation conditions. Because Sim4 has the largest network size among the 10 simulated conditions and is therefore the most computationally demanding, empirical runtime measurements on Smith Sim4 dataset (N = 50, T = 200, S = 50) are provided in Table S2. At the level of individual simulation conditions, MSTCM demonstrated particularly strong and consistent performance under demanding scenarios. In Sim4 (large‐scale network, N=50), MSTCM achieved AUC = 0.96±0.01, compared with 0.82±0.03 for the next best method (lsGC). In Sim6 (global mean confound), MSTCM achieved AUC = 0.88±0.03, compared with 0.80±0.08 (MCA‐LM). In Sim9 (strong connections), MSTCM achieved AUC = 0.88±0.02, compared with 0.81±0.06 (CCS). In Sim5 (shared inputs), MSTCM achieved AUC = 0.85±0.06, compared with 0.75±0.11 (CCS). Under Sim10 (nonstationary connections), MSTCM achieved AUC = 0.78±0.11, comparable to the next best method MCA‐LM (0.77±0.09), indicating that performance under nonstationary conditions remains a challenge for all methods evaluated.

On the overall aggregated data (All), MSTCM significantly outperformed all baseline methods: its AUC was 0.88±0.07, a 14.64% improvement over the next best method; its MCC was 0.55±0.09, substantially higher than other methods; its precision was 0.48±0.04, 1.79 times the average precision of the baseline methods, and its standard deviation was only 20.96% of the average standard deviation of the baseline methods; sensitivity reached 0.86±0.16, a 12.50% improvement over the next best method (p<0.001 for all four evaluation metrics).

### 3.2 taVNS Experiment Results

For all taVNS‐related analyses, within‐group and between‐group statistical analyses were performed. The functional connectivity and effective connectivity identified in this study exhibited non‐Gaussian distributions (consistent with classical findings that BOLD signals are non‐Gaussian due to the Gamma‐distributed nature of cerebrovascular hemodynamics (Hanson and Bly 2001), spatial heterogeneity in voxel variability and head motion (Chen et al. 2003), and asymmetric Rician distribution of magnitude MR signals (Wink and Roerdink 2006)). For within‐group analyses, post‐stimulation values were compared with pre‐stimulation values using the Wilcoxon signed‐rank test (Wilcoxon 1945). To assess taVNS‐specific effects, change scores (Δ= post − pre) were computed for each participant and compared between the taVNS and Sham groups using the Mann–Whitney U test (Mann and Whitney 1947). Benjamini–Hochberg false discovery rate (FDR) correction was applied to all comparisons. Effect sizes were reported for all statistical comparisons, using matched‐pairs rank‐biserial correlation $r=Z/\sqrt{N}$ for within‐group Wilcoxon signed‐rank tests, and rank‐biserial correlation $r_{\mathrm{rb}}=2U/\left(n_{1}\times n_{2}\right)-1$ for between‐group Mann–Whitney U tests (Kerby 2014). Effect size tables are provided in Tables S3–S7.

#### 3.2.1 Regulatory Effects of taVNS on Functional Connectivity

**FIGURE 6:**
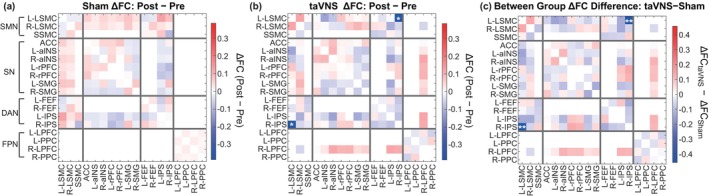
Changes in functional connectivity associated with taVNS. (a, b) Within‐group functional connectivity difference matrices for the Sham group and the taVNS group, respectively. In (a) and (b), red cells indicate increased connection strength after stimulation, whereas blue cells indicate decreased connection strength after stimulation (*p<0.05, Wilcoxon signed‐rank test with FDR correction). (c) Between‐group comparison of functional connectivity change scores. In (c), red cells indicate larger ΔFC values (Δ= post − pre) in the taVNS group than in the Sham group, whereas blue cells indicate smaller ΔFC values in the taVNS group than in the Sham group (**p<0.01, Mann–Whitney U test with FDR correction).

To evaluate the modulatory effects of taVNS on functional connectivity, we first computed the ROI‐wise functional connectivity matrices for both the Sham and taVNS groups, pre‐ and post‐stimulation, using the SM‐FC module. As shown in Figure 6, the results revealed that, in the Sham group, no significant differences were found in the functional connectivity between any ROI pairs. In contrast, the taVNS group exhibited a significant decrease in connection strength between the left lateral sensorimotor cortex (L‐LSMC) within the sensorimotor network (SMN) and the right intraparietal sulcus (R‐IPS) within the dorsal attention network (DAN) (p<0.05), suggesting that this cross‐network connection may be associated with the modulatory effects of taVNS. Between‐group comparison of stimulation‐induced change further confirmed that this reduction was significantly greater in the taVNS group than in the Sham group (p<0.01).

**FIGURE 7:**
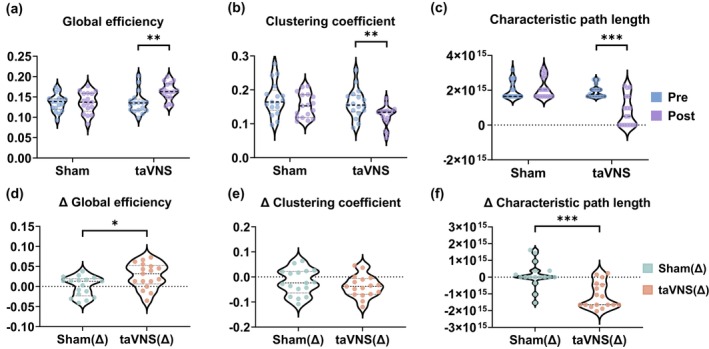
Changes in global brain network topological properties associated with taVNS. (a–c) Within‐group comparisons of global efficiency, clustering coefficient, and characteristic path length before and after stimulation in the Sham and taVNS groups, respectively. (d–f) Between‐group comparisons of stimulation‐induced change scores for global efficiency, clustering coefficient, and characteristic path length, respectively. In (a–c), significance was assessed using the Wilcoxon signed‐rank test with FDR correction. In (d–f), significance was assessed using the Mann–Whitney U test with FDR correction. (*p<0.05, **p<0.01, ***p<0.001).

To assess the effects of taVNS on the global topological properties of the brain functional network, we calculated and compared three global network metrics: global efficiency, clustering coefficient, and characteristic path length (Li, Yu, et al. 2021). As illustrated in Figure 7, the results revealed no significant changes in any of the metrics after stimulation in the Sham group. In contrast, the taVNS group showed significant alterations after stimulation across all three metrics: global efficiency significantly increased (p<0.01), whereas both the clustering coefficient (p<0.01) and the characteristic path length (p<0.001) significantly decreased. Between‐group comparisons of stimulation‐induced change further revealed significant group differences for global efficiency (p<0.05) and characteristic path length (p<0.001), but not for clustering coefficient (p=0.35), indicating that taVNS specifically increased global efficiency and reduced characteristic path length relative to Sham stimulation. The non‐significant between‐group difference in clustering coefficient suggests that the within‐group decrease observed in the taVNS group may not constitute a taVNS‐specific effect, and this finding should therefore be interpreted with caution. These findings suggest that taVNS modulates not only local functional connectivity between ROIs but also the global topological organization of the brain functional network.

#### 3.2.2 Regulatory Effects of taVNS on Effective Connectivity

**FIGURE 8:**
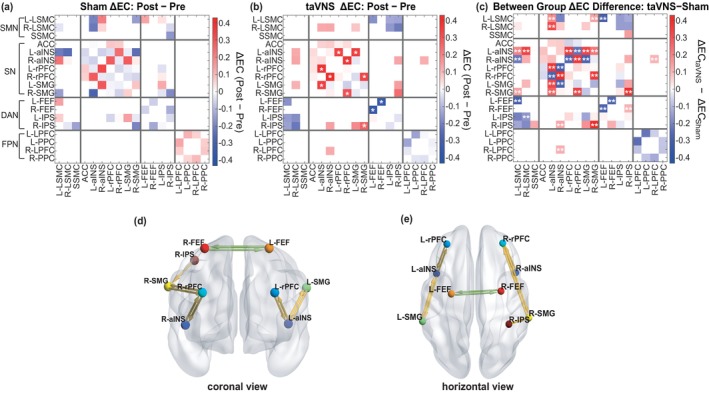
Changes in effective connectivity associated with taVNS. (a) Effective connectivity difference matrix for the Sham group (post − pre). (b) Effective connectivity difference matrix for the taVNS group (post − pre). The vertical axis denotes brain regions exerting causal influence, and the horizontal axis denotes brain regions receiving causal influence. In (a) and (b), red cells indicate increased directed connection strength after stimulation, whereas blue cells indicate decreased strength (*p<0.05, Wilcoxon signed‐rank test with FDR correction). (c) Between‐group comparison of stimulation‐induced EC change scores; red cells indicate larger ΔEC values (Δ= post − pre) in the taVNS group than in the Sham group, whereas blue cells indicate smaller ΔEC values in the taVNS group than in the Sham group (**p<0.01, Mann–Whitney U test with FDR correction). (d–e) Spatial distributions of core taVNS‐specific EC effects (connections significant in both within‐group and between‐group comparisons) shown in coronal and horizontal views, respectively. Yellow arrows indicate increased directed connection strength; green arrows indicate decreased strength.

To investigate the modulatory effects of taVNS on effective connectivity within brain networks, we employed the MSTCM algorithm proposed in this study to compare effective connectivity before and after stimulation in both the Sham and taVNS groups. As illustrated in Figure 8a,b, the results revealed that, in the Sham group, no significant differences were observed in effective connectivity after stimulation. In contrast, in the taVNS group, a total of 11 directed ROI‐to‐ROI connections exhibited statistically significant alterations (p<0.05). These significant connections were distributed within the salience network (SN) and DAN, and included bidirectional connections among supramarginal gyrus (SMG), rostral prefrontal cortex (rPFC), and anterior insula (aINS); long‐range bidirectional connections between the left and right frontal eye fields (FEF) across hemispheres; and a unidirectional connection from the right intraparietal sulcus (R‐IPS) to the right supramarginal gyrus (R‐SMG). Between‐group comparisons of stimulation‐induced change scores further revealed 34 directed edges with significant group differences after FDR correction (p<0.01). Importantly, all 11 within‐group significant connections in the taVNS group were confirmed by the between‐group comparison.

Figure 8d,e present the significant directed connections in the taVNS group before and after stimulation, visualized using the BrainNet Viewer toolbox (Xia et al. 2013). Specifically, within SN, the bidirectional connections from L‐aINS to L‐rPFC and from L‐aINS to L‐SMG were significantly enhanced. Concurrently, the bidirectional connections from R‐rPFC to R‐aINS and from R‐rPFC to R‐SMG were also enhanced. Within DAN, the bidirectional connections between L‐FEF and R‐FEF were significantly weakened. Furthermore, the unidirectional connection from R‐IPS in DAN to R‐SMG in SN increased significantly in strength. In summary, taVNS induced a marked reorganization of effective connectivity across brain networks after stimulation: information integration within the SN was significantly enhanced, integration within the DAN was significantly weakened, and directional information flow from the DAN to the SN was significantly enhanced. These findings suggest that taVNS modulates brain functional states by altering the information transmission mechanisms between the SN and DAN.

**FIGURE 9:**
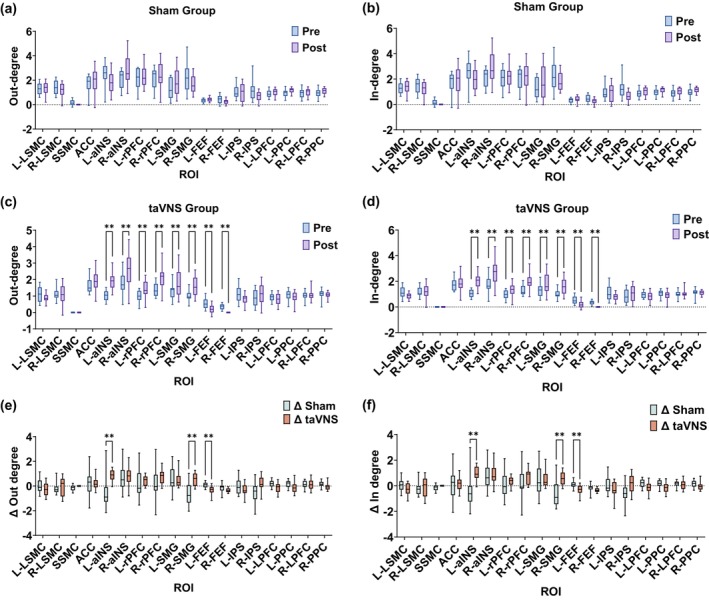
Changes in ROI out‐degree and in‐degree associated with taVNS. (a–b) Within‐group comparisons of out‐degree and in‐degree before and after stimulation in the Sham group. (c–d) Within‐group comparisons of out‐degree and in‐degree before and after stimulation in the taVNS group. (e–f) Between‐group comparisons of stimulation‐induced change for out‐degree and in‐degree, respectively. In (a–d), significance was assessed using the Wilcoxon signed‐rank test with FDR correction. In (e–f), significance was assessed using the Mann–Whitney U test with FDR correction (**p<0.01).

To further examine the modulatory effects of taVNS on information transmission among brain regions, we computed the out‐degree and in‐degree for each ROI at the individual level, both pre‐ and post‐stimulation, to assess changes in information output and reception within the network. As illustrated in Figure 9, no significant differences were observed in the Sham group, suggesting stable network connectivity patterns under Sham stimulation. In contrast, the taVNS group showed significant alterations in several key regions: the out‐degree and in‐degree of the bilateral aINS, rPFC, and SMG increased significantly (p<0.01), whereas those of the bilateral FEF decreased significantly (p<0.01). Between‐group comparisons of stimulation‐induced change further revealed significant group differences in L‐aINS, R‐SMG, and L‐FEF for both out‐degree and in‐degree after FDR correction. Specifically, L‐aINS and R‐SMG showed significantly greater increases (p<0.01) in the taVNS group relative to the Sham group, whereas L‐FEF showed significantly greater decreases (p<0.01). These findings indicate that taVNS modulates the information flow between brain regions at the node level, reflecting a pronounced regulatory effect on the brain network transmission mechanisms.

## 4 Discussion

### 4.1 Performance of the Proposed Model Considering Brain Modular Characteristics and Time‐Delay Effects

To address two key challenges in taVNS‐induced brain network modeling: (i) the limitation of single‐scale functional connectivity algorithms in distinguishing intra‐ and inter‐module connection patterns, and (ii) the instability of causal inference resulting from the low temporal resolution of fMRI signals, we propose the MSTCM algorithm. MSTCM integrates multiscale functional connectivity analysis with time‐delay‐sensitive causal modeling, thereby enhancing the stability of causal inference and improving its consistency with known neurophysiological mechanisms. Compared with conventional approaches, MSTCM is designed to align with the brain's small‐world architecture and hierarchical organization, enabling a more comprehensive representation of multilevel information interactions across brain networks. In simulated data, MSTCM exhibited superior robustness, and when applied to real fMRI data, it revealed cross‐network regulatory mechanisms that were difficult to detect using existing methods. Therefore, MSTCM not only provides methodological innovations but also serves as a powerful analytical algorithm for systematically investigating the neuroregulatory effects of taVNS.

### 4.2 Regulatory Effects at the Functional Connectivity Level

At the functional connectivity level, the taVNS group exhibited a significant reduction in functional connectivity between L‐LSMC and R‐IPS. L‐LSMC is primarily involved in motor execution and somatosensory feedback (Yeo et al. 2011), whereas R‐IPS plays a dominant role in regulating external attention (Santacroce et al. 2024). The decreased coupling between these two regions suggests a reduced synergistic interaction between exteroceptive processing and motor feedback. This observation is further corroborated at multiple levels of network analysis: at the effective connectivity level, causal connectivity among bilateral aINS, rPFC, and SMG nodes within the SN was significantly enhanced (p<0.01), with the aINS being a well‐established core interoceptive hub (Craig 2009; Critchley et al. 2004); at the node level, the out‐degree and in‐degree of L‐aINS showed significantly greater increases in the taVNS group relative to the Sham group (p<0.01), while those of L‐FEF (a core DAN node supporting exteroceptive attention) showed significantly greater decreases (p<0.01). Consistent with prior reports that vagus nerve stimulation suppresses sensorimotor cortex excitability (Gerges et al. 2025) and enhances interoception‐related neural activity (Paciorek and Skora 2020; Villani et al. 2019; Ventura‐Bort and Weymar 2024; De Smet et al. 2021; Richter et al. 2021), these converging findings collectively suggest a possible rebalancing of information processing toward interoceptive pathways.

At the global network topology level, taVNS significantly increased global efficiency and reduced characteristic path length, indicating enhanced information transmission efficiency across the brain network. This finding is consistent with the results of Guo et al. (Guo et al. 2024), who reported that taVNS improved emotional and cognitive function in patients with major depressive disorder, thereby providing a network‐level explanation for its clinical effects. Collectively, these results suggest that taVNS attenuates the coupling between external attention and motor feedback networks while facilitating more efficient brain‐wide communication, ultimately promoting improvements in emotional regulation and cognitive control.

### 4.3 Effects at the Effective Connectivity Level

At the effective connectivity level, taVNS significantly enhanced causal connections within the SN, while weakening the internal connections within the DAN and strengthening the directed information flow from DAN to SN. Within the SN, strengthened bidirectional interactions were observed among the bilateral aINS, rPFC, and SMG. The SN is known to integrate sensory, emotional, and cognitive information and to mediate switching between large‐scale brain networks (Schimmelpfennig et al. 2023; Stein et al. 2025; Snyder et al. 2021). The enhancement of intra‐network effective connectivity within the SN suggests that taVNS may improve cognitive flexibility and the efficiency of attentional resource allocation. In contrast, the DAN primarily supports the maintenance and allocation of goal‐directed attention (Alves et al. 2022; Sani et al. 2021). The observed reduction in intra‐network DAN connectivity, accompanied by increased information flow toward the SN, indicates an inhibition of sustained goal‐directed attention processes, thereby facilitating flexible network switching and attentional reallocation driven by the SN. These results are consistent with our functional connectivity findings and align with previous reports that vagus nerve stimulation enhances cognitive flexibility (Driskill et al. 2022; Naparstek et al. 2023).

The nodal out‐degree and in‐degree analysis further corroborated these findings. Following stimulation, the out‐degree and in‐degree of L‐aINS and R‐SMG increased significantly (p<0.01, respectively), indicating strengthened hub roles for these regions within SN. Conversely, both measures decreased in L‐FEF of the DAN (p<0.01), suggesting a diminished role in sustaining goal‐directed attention.

In summary, taVNS appears to promote a shift in information processing from exteroception toward interoception and to enhance cognitive flexibility by strengthening SN integration, attenuating DAN functionality, and modulating key hub nodes at the effective connectivity level. This mechanism provides a neuroimaging basis for understanding the clinical efficacy of taVNS in improving cognitive and emotional functions.

### 4.4 Limitations

Although the present analysis was intentionally focused on four predefined cortical networks, the proposed MSTCM framework is directly applicable to whole‐brain atlases and can be readily extended to additional systems such as the default mode network (DMN) and limbic networks. Future studies adopting a whole‐brain, atlas‐based ROI selection strategy would enable a more comprehensive characterization of taVNS‐induced network modulation across the whole brain. A further limitation concerns the absence of concurrent heart rate and respiratory recordings during fMRI acquisition, which precluded the application of RETROICOR‐based physiological noise correction. Although aCompCor was employed as a validated surrogate approach, future studies incorporating dedicated physiological monitoring would achieve superior physiological noise removal, allowing for a more comprehensive assessment of taVNS's neuromodulatory effects.

## 5 Conclusion

This study introduces a Multiscale Spatiotemporal Causal Mapping (MSTCM) algorithm that enhances the stability of fMRI‐based causal inference and provides empirical insights into the neural mechanisms through which taVNS modulates brain networks. At the functional connectivity level, taVNS facilitates a shift from exteroceptive to interoceptive processing and improves overall information transmission efficiency. At the effective connectivity level, taVNS strengthens integration within the SN while attenuating the dominance of the DAN. Together, these effects enhance cognitive flexibility and emotional regulation, offering new neuroimaging evidence supporting the clinical utility of taVNS. Moreover, the proposed MSTCM algorithm serves as both a methodological tool and a theoretical foundation for future investigations into neural regulation mechanisms and neuromodulation strategies.

## Funding

This work was supported in part by the National Natural Science Foundation of China under grant 62271141, and in part by the Fundamental Research Funds for the Central Universities under grant 2242023k30022.

## Supporting information


**Table S1:** Performance comparison of MSTCM and baseline causal inference methods across simulated fMRI datasets. Values are presented as mean ± standard deviation. Bold values indicate the best performance for each metric and condition.
**Table S2:**: Empirical runtime comparison of MSTCM and baseline methods on Smith Sim4 (N=50, T=200, S=50; five repeated measurements). Hardware: Intel i9‐14900HX, 32 GB RAM, Windows 64‐bit, MATLAB R2024a/Python.
**Table S3:**: Effect sizes for significant functional connectivity changes. 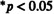; 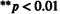 (Wilcoxon signed‐rank test or Mann–Whitney U test, FDR‐corrected); unmarked comparisons are non‐significant.
**Table S4:**: Effect sizes for significant changes in global topological properties. 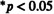; 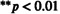; 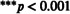 (Wilcoxon signed‐rank test or Mann–Whitney U test, FDR‐corrected); unmarked comparisons are non‐significant.
**Table S5:**: Effect sizes for significant effective connectivity changes. 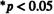; 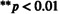 (Wilcoxon signed‐rank test or Mann–Whitney U test, FDR‐corrected); unmarked comparisons are non‐significant.
**Table S6:**: Effect sizes for significant changes in nodal out‐degree. 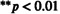 (Wilcoxon signed‐rank test or Mann–Whitney U test, FDR‐corrected); unmarked comparisons are non‐significant.
**Table S7:**: SEffect sizes for significant changes in nodal in‐degree. 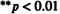 (Wilcoxon signed‐rank test or Mann–Whitney U test, FDR‐corrected); unmarked comparisons are non‐significant.

## Data Availability

The data that support the findings of this study are available from the corresponding author upon reasonable request.
