# Supplementary material for: A Multiscale Spatiotemporal Causal Mapping Algorithm for Revealing Neural Network Mechanisms of Transcutaneous Auricular Vagus Nerve Stimulation
Source: Hum Brain Mapp. 2026 Jul 22;47(11):e70615. doi: 10.1002/hbm.70615 (PMC13392224; doi:10.1002/hbm.70615)
Supplement: Supplementary file 1 — Table S1: Performance comparison of MSTCM and baseline causal inference methods across simulated fMRI datasets. Values are presented as mean ± standard deviation. Bold values indicate the best performance for each metric and condition. Table S2:: Empirical runtime comparison of MSTCM and baseline methods on Smith Sim4 (N=50, T=200, S=50; five repeated measurements). Hardware: Intel i9‐14900HX, 32 GB RAM, Windows 64‐bit, MATLAB R2024a/Python. Table S3:: Effect sizes for significant functional connectivity changes. ; (Wilcoxon signed‐rank test or Mann–Whitney U test, FDR‐corrected); unmarked comparisons are non‐significant. Table S4:: Effect sizes for significant changes in global topological properties. ; ; (Wilcoxon signed‐rank test or Mann–Whitney U test, FDR‐corrected); unmarked comparisons are non‐significant. Table S5:: Effect sizes for significant effective connectivity changes. ; (Wilcoxon signed‐rank test or Mann–Whitney U test, FDR‐corrected); unmarked comparisons are non‐significant. Table S6:: Effect sizes for significant changes in nodal out‐degree. (Wilcoxon signed‐rank test or Mann–Whitney U test, FDR‐corrected); unmarked comparisons are non‐significant. Table S7:: SEffect sizes for significant changes in nodal in‐degree. (Wilcoxon signed‐rank test or Mann–Whitney U test, FDR‐corrected); unmarked comparisons are non‐significant. [file HBM-47-e70615-s001.pdf]

SUPPLEMENTARY MATERIAL

Table S1: Performance comparison of MSTCM and baseline causal inference methods across simulated fMRI datasets. Values are presented as mean ± standard deviation. Bold values indicate the best performance for each metric and condition.

Condition	Metric	lsGC	MVARp	MCA-LM	CCS	FPCMCI	SDCD	TSCI	MSTCM
Sim1	AUC	0.58 ± 0.13	0.48 ± 0.12	0.72 ± 0.12	0.78 ± 0.12	0.54 ± 0.07	0.76 ± 0.10	0.63 ± 0.15	**0.87 ± 0.06**
	Precision	0.37 ± 0.37	0.10 ± 0.11	0.38 ± 0.11	0.37 ± 0.15	0.42 ± 0.40	0.40 ± 0.21	0.29 ± 0.15	**0.50 ± 0.04**
	Sensitivity	0.18 ± 0.17	0.18 ± 0.22	0.66 ± 0.22	0.71 ± 0.25	0.45 ± 0.43	0.41 ± 0.20	0.56 ± 0.26	**0.84 ± 0.20**
	MCC	0.13 ± 0.26	−0.13 ± 0.17	0.32 ± 0.18	0.28 ± 0.25	0.13 ± 0.23	0.25 ± 0.25	0.16 ± 0.24	**0.53 ± 0.11**
Sim2	AUC	0.58 ± 0.09	0.58 ± 0.09	0.77 ± 0.07	0.77 ± 0.06	0.52 ± 0.03	0.80 ± 0.07	0.67 ± 0.08	**0.93 ± 0.03**
	Precision	0.28 ± 0.16	0.10 ± 0.07	0.22 ± 0.04	0.22 ± 0.07	0.29 ± 0.33	0.34 ± 0.14	0.18 ± 0.04	**0.47 ± 0.03**
	Sensitivity	0.27 ± 0.13	0.23 ± 0.19	0.74 ± 0.13	0.70 ± 0.21	0.27 ± 0.39	0.45 ± 0.16	0.65 ± 0.13	**0.85 ± 0.11**
	MCC	0.18 ± 0.13	0.01 ± 0.10	0.27 ± 0.08	0.25 ± 0.13	0.08 ± 0.14	0.29 ± 0.17	0.18 ± 0.09	**0.58 ± 0.05**
Sim3	AUC	0.65 ± 0.07	0.59 ± 0.08	0.79 ± 0.06	0.76 ± 0.05	0.52 ± 0.03	0.77 ± 0.06	0.67 ± 0.09	**0.91 ± 0.03**
	Precision	0.33 ± 0.13	0.12 ± 0.03	0.16 ± 0.02	0.16 ± 0.03	0.35 ± 0.37	0.23 ± 0.08	0.13 ± 0.03	**0.44 ± 0.03**
	Sensitivity	0.30 ± 0.13	0.43 ± 0.15	0.79 ± 0.11	0.72 ± 0.12	0.13 ± 0.27	0.39 ± 0.14	0.64 ± 0.12	**0.82 ± 0.09**
	MCC	0.25 ± 0.10	0.09 ± 0.07	0.25 ± 0.06	0.22 ± 0.05	0.10 ± 0.13	0.22 ± 0.12	0.14 ± 0.08	**0.55 ± 0.04**
Sim4	AUC	0.82 ± 0.03	0.63 ± 0.04	0.81 ± 0.03	0.77 ± 0.03	0.51 ± 0.01	0.78 ± 0.02	0.67 ± 0.04	**0.96 ± 0.01**
	Precision	0.24 ± 0.05	0.03 ± 0.00	0.04 ± 0.00	0.05 ± 0.01	0.21 ± 0.16	0.07 ± 0.01	0.04 ± 0.00	**0.44 ± 0.02**
	Sensitivity	0.50 ± 0.08	0.64 ± 0.06	**0.86 ± 0.05**	0.76 ± 0.06	0.03 ± 0.02	0.34 ± 0.06	0.69 ± 0.05	0.84 ± 0.04
	MCC	0.32 ± 0.05	0.06 ± 0.02	0.12 ± 0.02	0.13 ± 0.02	0.07 ± 0.05	0.11 ± 0.03	0.07 ± 0.02	**0.60 ± 0.02**
Sim5	AUC	0.60 ± 0.13	0.47 ± 0.13	0.73 ± 0.10	0.75 ± 0.11	0.54 ± 0.08	0.73 ± 0.11	0.66 ± 0.15	**0.85 ± 0.06**
	Precision	0.42 ± 0.37	0.15 ± 0.11	0.34 ± 0.08	0.38 ± 0.09	0.34 ± 0.34	0.34 ± 0.19	0.31 ± 0.11	**0.43 ± 0.04**
	Sensitivity	0.22 ± 0.20	0.32 ± 0.28	0.70 ± 0.23	0.77 ± 0.18	0.34 ± 0.38	0.38 ± 0.22	0.60 ± 0.25	**0.90 ± 0.17**
	MCC	0.18 ± 0.27	−0.06 ± 0.22	0.28 ± 0.15	0.36 ± 0.14	0.10 ± 0.23	0.19 ± 0.24	0.22 ± 0.20	**0.50 ± 0.08**
Sim6	AUC	0.58 ± 0.14	0.48 ± 0.11	0.80 ± 0.08	0.79 ± 0.08	0.53 ± 0.09	0.74 ± 0.10	0.63 ± 0.13	**0.88 ± 0.03**
	Precision	0.39 ± 0.34	0.10 ± 0.10	0.35 ± 0.07	0.38 ± 0.10	0.32 ± 0.39	0.36 ± 0.19	0.33 ± 0.14	**0.50 ± 0.02**
	Sensitivity	0.22 ± 0.20	0.18 ± 0.21	0.87 ± 0.17	0.80 ± 0.23	0.24 ± 0.33	0.40 ± 0.19	0.56 ± 0.25	**0.98 ± 0.05**
	MCC	0.15 ± 0.24	−0.14 ± 0.16	0.37 ± 0.13	0.37 ± 0.20	0.09 ± 0.24	0.20 ± 0.24	0.22 ± 0.21	**0.61 ± 0.04**
Sim7	AUC	0.57 ± 0.08	0.58 ± 0.08	0.74 ± 0.08	0.72 ± 0.08	0.51 ± 0.03	0.81 ± 0.06	0.63 ± 0.10	**0.93 ± 0.02**
	Precision	0.29 ± 0.20	0.11 ± 0.07	0.20 ± 0.04	0.20 ± 0.06	0.17 ± 0.27	0.33 ± 0.10	0.17 ± 0.05	**0.47 ± 0.03**
	Sensitivity	0.22 ± 0.11	0.24 ± 0.18	0.73 ± 0.13	0.68 ± 0.21	0.29 ± 0.43	0.44 ± 0.13	0.58 ± 0.18	**0.84 ± 0.13**
	MCC	0.16 ± 0.14	0.02 ± 0.10	0.23 ± 0.08	0.19 ± 0.13	0.03 ± 0.12	0.29 ± 0.12	0.15 ± 0.12	**0.57 ± 0.07**
Sim8	AUC	0.58 ± 0.11	0.45 ± 0.09	0.78 ± 0.08	0.81 ± 0.11	0.52 ± 0.06	0.79 ± 0.08	0.64 ± 0.14	**0.87 ± 0.04**
	Precision	0.31 ± 0.29	0.10 ± 0.09	0.37 ± 0.09	0.40 ± 0.16	0.31 ± 0.34	0.42 ± 0.14	0.33 ± 0.12	**0.49 ± 0.04**
	Sensitivity	0.18 ± 0.17	0.17 ± 0.19	0.72 ± 0.21	0.75 ± 0.25	0.44 ± 0.45	0.43 ± 0.12	0.58 ± 0.23	**0.87 ± 0.18**
	MCC	0.10 ± 0.20	−0.15 ± 0.13	0.33 ± 0.15	0.35 ± 0.20	0.06 ± 0.20	0.27 ± 0.16	0.23 ± 0.21	**0.54 ± 0.11**
Sim9	AUC	0.55 ± 0.10	0.44 ± 0.12	0.79 ± 0.05	0.81 ± 0.06	0.58 ± 0.09	0.80 ± 0.09	0.69 ± 0.07	**0.88 ± 0.02**
	Precision	0.25 ± 0.29	0.16 ± 0.10	0.30 ± 0.05	0.36 ± 0.08	0.41 ± 0.31	0.48 ± 0.20	0.30 ± 0.08	**0.50 ± 0.01**
	Sensitivity	0.13 ± 0.14	0.35 ± 0.25	0.91 ± 0.15	0.87 ± 0.13	0.36 ± 0.29	0.48 ± 0.20	0.67 ± 0.23	**1.00 ± 0.03**
	MCC	0.05 ± 0.21	−0.07 ± 0.20	0.31 ± 0.09	0.37 ± 0.11	0.18 ± 0.22	0.34 ± 0.24	0.22 ± 0.16	**0.61 ± 0.02**
Sim10	AUC	0.52 ± 0.09	0.37 ± 0.12	0.77 ± 0.09	0.73 ± 0.10	0.53 ± 0.06	0.73 ± 0.11	0.60 ± 0.15	**0.78 ± 0.11**
	Precision	0.26 ± 0.31	0.02 ± 0.06	0.40 ± 0.09	0.32 ± 0.09	0.37 ± 0.38	0.37 ± 0.21	0.30 ± 0.13	**0.50 ± 0.03**
	Sensitivity	0.20 ± 0.28	0.04 ± 0.11	0.69 ± 0.17	**0.73 ± 0.20**	0.42 ± 0.43	0.38 ± 0.22	0.49 ± 0.24	0.68 ± 0.20
	MCC	0.06 ± 0.22	−0.24 ± 0.09	0.36 ± 0.13	0.27 ± 0.17	0.10 ± 0.21	0.21 ± 0.26	0.16 ± 0.21	**0.46 ± 0.11**
All	AUC	0.60 ± 0.13	0.51 ± 0.13	0.77 ± 0.08	0.77 ± 0.09	0.53 ± 0.06	0.77 ± 0.09	0.65 ± 0.12	**0.88 ± 0.07**
	Precision	0.31 ± 0.28	0.10 ± 0.09	0.28 ± 0.13	0.28 ± 0.15	0.32 ± 0.34	0.33 ± 0.19	0.24 ± 0.14	**0.48 ± 0.04**
	Sensitivity	0.24 ± 0.19	0.28 ± 0.25	0.77 ± 0.18	0.75 ± 0.20	0.30 ± 0.38	0.41 ± 0.17	0.60 ± 0.21	**0.86 ± 0.16**
	MCC	0.16 ± 0.21	−0.06 ± 0.17	0.28 ± 0.13	0.28 ± 0.17	0.09 ± 0.19	0.24 ± 0.21	0.18 ± 0.17	**0.55 ± 0.09**

Table S2: Empirical runtime comparison of MSTCM and baseline methods on Smith Sim4 ($N = 50$, $T = 200$, $S = 50$; 5 repeated measurements). Hardware: Intel i9-14900HX, 32 GB RAM, Windows 64-bit, MATLAB R2024a / Python.

**Algorithm**	**Runtime (mean ± SD, s)**	**Language**
lsGC	3.54 ± 0.10	MATLAB
MVARp	7345.25 ± 98.32	MATLAB
MCA-LM	2.99 ± 0.17	MATLAB
CCS	7446.03 ± 30.82	MATLAB
FPCMCI	1574.58 ± 37.51	Python
SDCD	1015.64 ± 160.28	Python
TSCI	797.89 ± 16.58	Python
MSTCM	144.28 ± 1.21	MATLAB

Table S3: Effect sizes for significant functional connectivity changes.

**Connection**	**taVNS (within-group r)**	**Sham (within-group r)**	**Between-group (r_{rb})**
L-LSMC ↔ R-IPS	0.88^{*}	−0.21	-0.88^{**}

$^{*}p < 0.05$; $^{**}p < 0.01$ (Wilcoxon signed-rank test or Mann–Whitney U test, FDR-corrected); unmarked comparisons are non-significant.

Table S4: Effect sizes for significant changes in global topological properties.

**Metric**	**taVNS (within-group r)**	**Sham (within-group r)**	**Between-group (r_{rb})**
Global efficiency	0.67^{**}	0.05	0.45^{*}
Clustering coefficient	-0.67^{**}	−0.37	−0.19
Characteristic path length	-0.82^{***}	0.15	-0.74^{***}

$^{*}p < 0.05$; $^{**}p < 0.01$; $^{***}p < 0.001$ (Wilcoxon signed-rank test or Mann–Whitney U test, FDR-corrected); unmarked comparisons are non-significant.

Table S5: Effect sizes for significant effective connectivity changes.

**Directed connection**	**taVNS (within-group r)**	**Sham (within-group r)**	**Between-group (r_{rb})**
L-aINS → L-rPFC	0.84^{*}	−0.22	0.75^{**}
L-rPFC → L-aINS	0.84^{*}	−0.10	0.76^{**}
L-aINS → L-SMG	0.87^{*}	−0.18	0.74^{**}
L-SMG → L-aINS	0.88^{*}	−0.02	0.72^{**}
R-aINS → R-rPFC	0.88^{*}	−0.10	0.76^{**}
R-rPFC → R-aINS	0.88^{*}	−0.12	0.80^{**}
R-rPFC → R-SMG	0.87^{*}	−0.17	0.80^{**}
R-SMG → R-rPFC	0.88^{*}	−0.25	0.79^{**}
L-FEF → R-FEF	-0.88^{*}	−0.22	-0.76^{**}
R-FEF → L-FEF	-0.88^{*}	−0.24	-0.74^{**}
R-IPS → R-SMG	0.76^{*}	−0.82	0.88^{**}

$^{*}p < 0.05$; $^{**}p < 0.01$ (Wilcoxon signed-rank test or Mann–Whitney U test, FDR-corrected); unmarked comparisons are non-significant.

Table S6: Effect sizes for significant changes in nodal out-degree.

**ROI**	**taVNS (within-group r)**	**Sham (within-group r)**	**Between-group (r_{rb})**
L-aINS	0.87**	−0.58	0.83**
R-aINS	0.75**	0.55	0.17
L-rPFC	0.73**	0.13	0.36
R-rPFC	0.78**	0.13	0.39
L-SMG	0.71**	0.48	−0.04
R-SMG	0.72**	−0.61	0.75**
L-FEF	−0.81**	0.33	−0.70**
R-FEF	−0.88**	−0.45	−0.45

** $p < 0.01$ (Wilcoxon signed-rank test or Mann–Whitney U test, FDR-corrected); unmarked comparisons are non-significant.

Table S7: Effect sizes for significant changes in nodal in-degree.

**ROI**	**taVNS (within-group r)**	**Sham (within-group r)**	**Between-group (r_{rb})**
L-aINS	0.86**	−0.57	0.82**
R-aINS	0.82**	0.66	0.13
L-rPFC	0.73**	0.10	0.32
R-rPFC	0.76**	0.14	0.42
L-SMG	0.69**	0.38	0.02
R-SMG	0.76**	−0.49	0.63**
L-FEF	−0.78**	0.30	−0.63**
R-FEF	−0.88**	−0.53	−0.47

** $p < 0.01$ (Wilcoxon signed-rank test or Mann–Whitney U test, FDR-corrected); unmarked comparisons are non-significant.
